# Abortion care over 20 weeks gestation: a psychosocial exploration of patient experiences at the Royal Women’s Hospital, Victoria, Australia

**DOI:** 10.1186/s12978-025-02179-8

**Published:** 2025-11-12

**Authors:** C. McDonald, C. Hannon, P. Moore, AN Wilson

**Affiliations:** 1 Abortion and Contraception Service, Royal Women’s Hospital, Parkville, VIC Australia; 2 Women’s, Children’s and Adolescent’s Health Program, International Development, Burnet Institute, Melbourne, VIC Australia; 3 Melbourne School of Population and Global Health, University of Melbourne, Melbourne, VIC Australia; 4 School of Public Health and Preventive Medicine, Monash University, Melbourne, VIC Australia

**Keywords:** Abortion, Abortion access, Termination, Thematic analysis, Barriers and enablers to abortion care

## Abstract

Abortion is legal in every state of Australia, yet access to abortion over 20 weeks gestation is not universally guaranteed. Patients with indications outside of maternal medicine or fetal abnormalities experience particular challenges, including stigmatisation and limited service access. The aim of this study was to explore the lived experiences of women accessing abortion care over 20 weeks at the Royal Women’s Hospital for non-medical reasons, identify enablers and barriers to care and understand experiences of abortion care. Participants were recruited via convenience sampling once their abortion care had been booked. Semi-structured interviews were conducted in person or online. Data was analysed via a reflexive thematic analysis. 15 women participated in the study. A total of five themes and 15 sub-themes were identified which described social stressors and systemic barriers to abortion access, including multiple competing personal stressors, compromised autonomy, navigating a complex abortion service system and late diagnosis of pregnancy as compounding reasons for delayed access to abortion care. Enablers to abortion care recognised the importance of safe, holistic, and time-sensitive care for all. Additional research is needed to understand the experiences of women who attempted to access abortion care over 20 weeks but were unable to do so and forced to continue their pregnancies. Greater access to abortion at 20 weeks and over for reasons beyond maternal medicine and fetal abnormalities is needed throughout Victoria and Australia-wide.

## Background

Despite abortion being legal in every state in Australia, there is a lack of adequate clinical services for abortion; publicly funded hospitals providing maternity and women’s health services often have limited or no abortion care available [1]. The limited access to abortion care women and pregnant people face can result in extended wait times, prolonged travel times and additional costs. Delays in time sensitive and appropriate health care can cause trauma for the women and pregnant people attempting to access care [2]. Psychosocial reasons for seeking an abortion at any gestation include socioeconomic disadvantage, reproductive control over childbearing, relationship issues, family violence, sexual assault, illicit drug use, mental health issues and late diagnosis of pregnancy [3–6]. The COVID-19 pandemic created additional systemic barriers to pre-existing abortion access challenges, particularly for women in marginalised and at-risk groups including refugee and migrant groups, international students, and Aboriginal and Torres Strait Islander people [1]. Previous Australian research has identified three primary barriers to abortion access - financial limitations, geographic barriers and deficiencies in practitioner attitudes, education and training [1].

Abortion is lawful in many countries, however abortion access can be restricted by varying forms of unique regulation and legislation [7]. Socio-cultural norms, including gender-binaries contribute to abortion stigma, which can manifest as abortion exceptionalism in health care systems, medical institutions, and individual attitudes towards abortion [8]. Generally, abortion is a common, safe, and non-complex healthcare intervention with low complication rates [7]. However, 45% of abortions worldwide are estimated to be unsafe with limited access to safe quality abortion care, resulting in women and girls being forced to seek unsafe and unskilled clinical services to end their pregnancies [7].

Abortion over 20 weeks gestation is especially stigmatised and associated with specific access difficulties relating to limited providers of advanced gestation abortion care [9]. Psychosocial or non-medical reasons women have abortions later in pregnancy may relate to concerns around existing or future children, commitments and responsibilities, lack of social support, lack of financial or emotional resources and perceived abortion stigma [5]. Competing social stressors such as family violence, unemployment, financial hardship, lower educational attainments, being an adolescent or young woman and needing to travel to obtain care are cited as compounding reasons for delays in accessing care [10, 11]. In 2018, 3132 non-British resident women travelled to England and Wales to access abortion services; approximately 95% of those women did so from the Republic of Ireland prior to the 2018 modification of the abortion law and the rest travelled from Italy, France, Germany and Denmark [12]. In the United States, half of women at reproductive ages describe living in states with ‘hostile’ abortion rights which increases the need to travel to access abortion care. Women of colour were found to be less likely to travel than white women [13]. Women who are forced to travel to access abortion care are subjected to delayed health care for a time-sensitive procedure as well as resourcing themselves with funding to travel to a service provider than can accommodate their abortion request.

Gestational age limits for abortion care further restricts women’s choices and compromises bodily autonomy when facing psychosocial circumstances often associated with abortion requests in the second trimester [14].

Abortion over 20 weeks gestation is stigmatised and associated with particular access difficulties relating to limited providers of advanced gestation abortion care [9]. The 2023 National Senate Inquiry into universal access to reproductive health care recommended that all public hospitals within Australia provide access to surgical terminations to ensure timely and affordable local pathways, as well as undertake efforts to improve access in regional areas [2].

In Victoria, Australia, the Abortion and Contraception Service (ACS) at the Royal Women’s Hospital (RWH) is the sole provider of abortion care over 20 weeks gestation for non-medical reasons in the state. This service is accessible for women and pregnant people with complex psychosocial and/or medical needs and/or who may have exceeded gestational age limits at local abortion services. The ACS is also one of few abortion providers for terminations over 20 weeks in Australia. The term ‘psychosocial’ is used to describe the relationship between intrapersonal, psychological and environmental influence on an individual’s mental health and behaviour [15]. Psychosocial reasons for obtaining abortion care, relate to social determinants of health including financial insecurity and lower educational attainment, which compound socio-economic and gender inequities [10]. Abortion care at later gestations, particularly for non-medical indications, remains significantly under-researched. Existing literature and public debate often focus on medical or fetal indications, with limited reference to the psychosocial reasons that delay access to care. This gap in knowledge perpetuates a narrow understanding of abortion and reinforces stigma. The aim of this study is to explore the lived experiences of women accessing abortion care over 20 weeks at the RWH, for non-medical indications and identify enablers and barriers to abortion care. Ethics approval was obtained from the Royal Women’s Hospital Human Research Ethics Committee (Project ID: 79615) to conduct this study.

## Methods

### Study design and setting

A qualitative study was conducted at the Abortion and Contraception Service (ACS) at the Royal Women’s Hospital (RWH), Victoria, Australia. The ACS team is a multidisciplinary team that includes consulting doctors, surgeons, nurses, midwives, social workers, Aboriginal health officers, administrative staff and psychiatrists. Within the ACS, approximately 12 abortions over 20 weeks gestation occur each month, with at least one abortion over 24 weeks gestation, for reasons outside of maternal health or fetal anomalies.

The thematic findings are presented as five themes and fifteen sub-themes (Table 1). Quotations in italics are taken verbatim from de-identified participant interview transcripts (e.g. P1-P15). Square brackets are used to clarify meaning and ellipses to condense a quotation.

All consent forms were stored securely in a password-protected computer file that only primary researchers in this study had access to.

**Table 1:** Participant characteristics

Participant characteristics		*N* = 15 *n* (%)
Age (years)	20–29 30–39	10 (66.67%) 5 (33.33%)
Country of Birth	Australia Fiji Malaysia Myanmar Pakistan Sri Lanka	9 (56.25%) 1 (6.25%) 1 (6.25%) 1 (6.25%) 1 (6.25%) 2 (12.5%)
Language other than English	Yes No	7 (43.75%) 8 (56.25%)
Lives in rural/regional area	Yes No	5 (33.33%) 10 (66.67%)
Education	High School not completed High school completed Certificate/Diploma Undergraduate degree Postgraduate	2 (13.33%) 2 (13.33%) 1 (6.67%) 9 (60%) 1 (6.67%)
Employment	Centrelink Student (Full-time) Student (Part-time)/Working Full-time work	4 (26.67%) 1 (6.67%) 3 (20%) 7 (46.67%)
Disability	Yes No	1 (6.25%) 14 (93.75%)
Pregnancy history	Nulliparous Multiparous	7 (43.75%) 8 (56.25%)
Gestational age at confirmation (weeks)	< 10 10–20 20+	5 (33.33%) 6 (40%) 4 (26.67%)
Gestational age at termination (weeks)	20–23 24 and over	11 (73.33%) 4 (26.67%)
Late diagnosis of pregnancy (where abortion < 20 weeks not possible)	Yes No	8 (56.25%) 7 (43.75%)
Concealed pregnancy	Yes No	8 (56.25%) 7 (43.75%)
Ambivalent in decision making	Yes No	7 (43.75%) 8 (56.25%)
Secure housing	Yes No	14 (93.33%) 1 (6.67%)
Housing type	Own Rental Short-term Homeless	4 (26.67%) 8 (53.33%) 2 (13.33%) 1 (6.67%)
Existing or history of mental health issues	Yes No	7 (46.67%) 8 (53.33%)
Current intimate partner violence	Yes No	4 (26.67%) 11 (73.33%)

### Participants and recruitment

Study participants were recruited according to inclusion criteria. Participants were eligible to take part in the study if they accessed an abortion at or over 20 weeks gestation and were over 18 years old. In addition, there could be no ethical concerns regarding the participant’s capacity to consent to the research project. Participants were approached by three social workers within the ACS, to participate in the study face-to-face following their initial clinic consultation, once consent for an abortion had been obtained and a procedure date confirmed. This order was purposive and acted to reinforce verbal communication that participation was voluntary and would have no impact on their abortion care. Participants were provided with a plain language statement to outline study information, and the primary aims and objectives of the research project. ACS social workers obtained written consent to participate in the study by the primary researchers. All researchers conducting the interviews were female identifying clinicians who worked or had worked as part of the ACS team which participants were aware of at the time of interview.

### Data collection

The interview guide was piloted and reviewed on several occasions before finalising the interview questions. Semi-structured interviews were completed one-on-one with participants either face-to-face or via online video conferencing (Zoom) by members of the research team, experienced trauma-informed clinicians, including social workers and a nurse/midwife. No interviewers were directly involved in the patient’s care. The distress protocol was referred to before each interview, making the participant aware of support available. None of the participants became distressed to the point of the interview prematurely ending or seeking additional support guided by the distress protocol. Interviews were semi-structured allowing for participants and interviewers to expand on areas of interest, particularly focusing on the lived experiences of women accessing abortion care and the barriers and enablers they encountered. Interviews were completed between April to December 2022, at a time and date that was convenient for the participant, including accommodating preferences for interviews to take place before or after their abortion date. All interviews focused on enablers and barriers to abortion care access beyond 20 weeks, rather than the experience of the abortion procedure itself. Interviews were conducted until there was sufficient information power to answer the research question in line with our interpretive approach [16]. Face-to-face interviews were conducted in a private interview room at RWH. A $50 voucher was provided to participants as a contribution for their time. Interview durations were between 40 and 60 min and recorded using a handheld recording device and/or via the Zoom recording function. Field notes were taken by interviewers and clarified with participants during and after the interviews. Transcripts were not returned to participants for comment or correction and were analysed verbatim by the lead researcher (CMD).

### Data analysis

Following the completion of the recruitment and interview process, interviews were then transcribed verbatim using Otter artificial intelligence transcription software [17]. The lead researcher reviewed and coded each interview for consistency and accuracy. The lead researcher de-identified each transcript to maintain confidentiality and replaced with participant identifying numbers (e.g. P1, P2). Data analysis was managed using Nvivo software [18]. Braun and Clarke’s six recursive steps of reflexive thematic analysis were used to analyse the data [19] familiarisation, coding, generating initial themes, reviewing, and developing themes, refining, defining, and naming themes and writing up [19].

Analysis and familiarisation with the transcripts involved the researcher listening to the transcript audio and reading the accompanying text thoroughly line-by-line to establish initial themes, ideas, and concepts from the data set. An initial set of codes were reviewed to ensure all relevant themes, ideas and concepts were captured. Domains explored in the interview guide related to positive and negative experiences in access to abortion care, changes in life circumstances, social and economic precarity and the impact of pregnancy diagnosis on emotional and practical levels. Transcripts were coded with relevance to enablers and barriers to abortion access, including the social context and personal circumstances of the women interviewed, such as housing instability, current family violence and financial insecurity. A co-researcher coded a subset of transcripts and provided regular input throughout the coding process, initial codes were reviewed and adapted through discussions between the primary researchers and the wider research team. Transcripts were regularly reviewed throughout the coding process to ensure consistency of the data collection. Primary researchers reviewed the dataset and codes to develop emerging themes, trends and findings from data interpretation and analysis. Codes, sub-themes and themes were refined through study team discussions.

Researchers considered questions of reflexivity by reflecting on their own biases and preconceptions of abortion care accessibility. Researchers acknowledged their strong support of reproductive health, abortion accessibility and lived experience of providing abortion care influenced the interpretation of the results. Researchers considered their familiarity of this context as useful to contextualise findings and interpret results, while maintaining a non-judgemental standpoint to participants’ views and opinions that deviated from their own.

## Results

### Participant characteristics

In total, 17 participants were recruited to engage in this study. However, one participant was excluded due to poor audio quality which was unable to be transcribed, and another was excluded due a delayed confirmation of gestational age on a formal scan. Ultimately, 15 patients participated. Patient demographics (Table 2) identified that just under half lived with existing or historic mental health issues and the majority reported living free from family violence and without a disability. Almost half of the participants were born overseas, with English being their second language. More than a third of participants were international students, studying or had recently completed their studies and were either in Australia on student or graduate visas. Over half of the women in this study concealed their pregnancy and described a late diagnosis of pregnancy. Concealed pregnancy is a complex, multidimensional and temporal process where a woman is aware of her pregnancy but chooses not to disclose on the basis of fear or perceived lack of support [20]. Most participants lived in metropolitan areas and accessed abortion care between 20 and 23 weeks’ gestation. Almost half of the participants in this study disclosed some ambivalence in their decision making to have an abortion at some point in their pregnancy. Most participants in this study were formally educated with tertiary qualifications, had stable employment and secure housing. No Aboriginal or Torres Strait Islander participants were included in this study.

**Table 2:** Themes and subthemes

Themes	Sub-themes
Multiple competing personal and social stressors	• Psychosocial reasons – Alcohol and other drugs (AOD), Mental health issues (MH), Housing instability, Financial hardship, Family Violence • Changes to life circumstances i.e. relationship breakdown, changes to housing and financial security • Impact of pregnancy diagnosis – emotional (signs of grief, loss, practical response in decision making, assessing financial situation and existing caring responsibilities)
Feeling of compromised autonomy	• Relationship breakdown including conflicting views with partner in decision making process and intimate partner violence and abuse – Family Violence (FV) present in current relationship or life or history of FV • Perceived/experience of coercion including feeling isolated in decision making (concealed pregnancy from Partner in pregnancy (PIP)/family in fear of judgement/lack of support) and systemic coercion to have an abortion (TOP) (i.e. influence of statutory agency (e.g. child protection) in personal decision making) • Concealed pregnancy – cultural stigma, fear of judgement and social isolation if pregnancy is discovered
Beyond health literacy	• Late diagnosis of pregnancy – impact of pregnancy diagnosis • Bodily attunement – no signs or symptoms of pregnancy • Medicare ineligible/international patient – navigating a health system in a new country
Navigating the complex abortion service labyrinth	• Systemic Barriers –primary health care barriers causing additional delays in accessing time-sensitive care (GP wait times, ultrasound wait times and delays in referral to a service that can provide abortion care) • Access to health information – self-advocacy, being pro-active in becoming an expert in abortion access in Victoria or passive health seeking information within a time limited context • Women seeking abortion shuffled between services (multi-referral process leading to feelings of disempowerment)
Enabling safe, timely, holistic and inclusive care for all	• Positive and negative experiences with health care providers • Importance of having a non-judgemental and supportive abortion o experience • Access uncertainty – feeling ‘lucky’ to access an abortion

### Multiple competing personal and social demands and stressors affect pregnancy decision making

This theme explores the life context of the participants and personal circumstances that influenced their decisions to seek abortion care. Participants described multiple psychosocial factors that influenced their pregnancy decision-making processes, and this prevented earlier engagement with abortion services. Psychosocial stressors were not experienced in isolation and impacted their personal, social, cultural, and economic status before an unplanned or unwanted pregnancy was confirmed.

#### Psychosocial stressors

Psychosocial stressors identified by participants included financial stress, struggling to cope with existing children, being a sole parent, insufficient support to continue the pregnancy, mental health issues, visa insecurity and housing instability. One participant described the difficulty of being a single parent *“I’m finding it hard enough to raise them [my children] and my concern is that being completely on my own with no support*,* no help and being backed into a corner where I could end up in a deep deep depression and that’s happened to me before. Because I know what it’s like having a newborn*,* I’ve had four” (P2).* The impact of these competing stressors and demands posed barriers to earlier presentations and were a driving factor in seeking an abortion. One participant described the emotional toll of competing stressors in her life, “*making me so much depressed…like everything my kids*,* my home*,* my financial status my visa everything is like just a mess” (P15).* Many women’s decision making in this study was influenced by anticipating their social situation worsening if they continued their pregnancy “*the social and financial impacts that would have had on me. I would lose my housing; I would have to quit my job. I would have…I have little money as is. Also*,* the aspect of the other partner in the pregnancy*,* that relationship…not a good one” (P10).*

#### Changes to life circumstances

Changes to life circumstances, such as relationship breakdowns, changes to housing and financial security or the loss of previous sources of support was another psychosocial reason in pregnancy decision making in this study. Many participants were not afforded the opportunity to prioritise their pregnancy decision making due to the urgency in managing life changes. As one participant described,

‘*I was dealing with homelessness and struggling with…going through a breakup with my ex-partner. Just resources as well. I lost my license. So*,* it kind of everything just happened all at once for me. So*,* I guess I just was really wrapped up in feeling sorry for myself and just didn’t focus on the fact that I was pregnant…I just kind of swept under the rug as much as I could until*,* yeah*,* it was really almost too late” (P11).*

Women experiencing abortion over 20 weeks gestation in this study demonstrated how they were unable to prioritise their pregnancies due to complexities in their lives with sudden life changes. One participant described how multiple factors had contributed to her decision making,

“*There are multiple reasons that I came to this point. It’s not like I can tell you a certain one. I have a financial problem. I have a visa problem. I have a funding problem [state funding for child with additional needs]. And these are the main concerns that I am going for the abortion” (P15).*

#### Impact of pregnancy diagnosis: ambivalence, emotional and practical response in decision making

The impact of pregnancy diagnosis varied across the participants in this study. Many were unaware of their pregnancies until they were in a position where an abortion earlier than 20 weeks was impossible. As one participated noted, “*It was really scary at the beginning*,* when I thought*,* Oh*,* my God*,* like*,* would I not be able to get an abortion?” (P1).* Responses to pregnancy diagnosis varied from emotional to practical responses, as well as ambivalence. One woman described her distress following her late diagnosis of pregnancy and despite making a decision on a practical level, she was deeply impacted by the emotional experience of proceeding with an abortion,

*“Oh my God*,* no*,* this is like my baby and I’ve never had to go through anything like this before because this has to be the worst thing ever that’s happened to me…But I would say it’s for the best I think for the baby*,* even though it’s not for me*,* because it will take a while for me to completely move on from this” (P8).*

Almost half of study participants disclosed uncertainty in their pregnancy decision making at some stage in their pregnancy. One woman felt unclear in her decision making for five months before coming to her decision and proceeding with an abortion request, *“Yeah*,* it [my decision] was very just always*,* up and down consistently” (P3).* Whereas other women were clear in their decision and based their decision on practicalities of their current life circumstances, as one woman outlined “*It was immediate. There was no question…No*,* in reality*,* I had made up my mind the second that I knew and I just wanted it out then and there” (P17).*

### Compromised autonomy

Participants in this study disclosed varying degrees of compromised autonomy, where the woman’s decision making was limited as a result of intimate partner violence and abuse, conflicting views with their partner or feeling disempowered by a lack of support in reaching a decision.

#### Relationship issues

Women experiencing relationship issues had conflicting views with their partners, which delayed earlier presentations for abortion care. One participant explained, “*He was clear of keeping the baby. I wasn’t” (P3).* Participants in the study who had contentious views with their partner, also referred to experiencing a lack of support from them. As one woman described, “*Yeah*,* my ex-partner who got me pregnant…he is not that kind of supportive person and a man who would like to take responsibility” (P6).* Other participants experienced current or recent intimate partner violence and abuse causing an ongoing sense of fear, attributing to the decision to have an abortion. One participant described, *“The father [of the baby] was abusive…a family violence incident did occur*,* where he was removed from the property” (P2).* Participants who experienced volatile and abusive relationships were left to go it alone, knowing support from the PIP was not available to them and therefore choosing not to disclose the pregnancy.

#### Perceived and experience of coercion

Many participants in the study reported a sense of isolation in their pregnancy decision making process, feeling unable to disclose their pregnancy to anyone in fear of being judged, misunderstood, or coerced into a decision that was not their own. As one woman noted “*They [PIP] Don’t even know…I didn’t even tell them” (P2).* One participant experienced a significant amount of child protection involvement with her existing children and felt the statutory influence impacted her personal decision making. This influence could be perceived as systemic coercion, where the woman’s autonomy was compromised, “*So*,* if we decide to have a child*,* we’ve got to deal with all this. We have to deal with them [child protection] getting involved all of the time” (P2).*

#### Concealed pregnancy

Participants who concealed pregnancies cited fears of judgement or cultural shame for being pregnant outside of marriage. As one woman described, *“I didn’t want to talk about this [pregnancy] with anyone. Because I felt like I’ll be judged in a certain way” (P8).* Women who concealed their pregnancies reported feeling isolated and lacking support. One participant described, *“Where do I turn to now? I just wanted one person to understand” (P2).* Participants who concealed their pregnancies from family overseas feared judgement and disengaged from contact with family members, contributing to feelings of guilt, shame, and social isolation,

“*I didn’t really want to talk to my family members at that time as well*,* because they don’t know anything about it…I feel guilty. Yeah*,* to hide this from my family members who are very good to me… I’m not feeling myself… I didn’t really call them for a while. I used to talk to them every two days…and text them every day”. (P6).*

### Beyond health literacy

Women’s descriptions of their experiences indicated varying degrees of sexual and reproductive health literacy resulting in late diagnosis of pregnancy, challenges for international patients navigating a foreign health system and geographical location determining time-sensitive care.

#### Late diagnosis of pregnancy

Most women had their pregnancies confirmed at a later stage in pregnancy, which meant they were unable to consider an abortion at an earlier gestation. A participant described her shock at discovering her pregnancy at an advanced gestation when she had had no prior signs or symptoms of pregnancy, (P12) “*Never once thought that could be pregnant*,* especially in the first few months*,* when*,* you know*,* you typically hear about morning sickness*,* and sore breasts and things. I think that it was a lack of*,* personally*,* me having never been pregnant for I don’t know what the feelings are like”.* Other women in this study presented with poor health literacy in the context of contraception use and sexual education, *“I didn’t use any contraception*,* but I just thought oh*,* it’s not going to happen to me” (P6).*

#### International patients navigating a foreign health system

Women from culturally and linguistically diverse backgrounds described additional challenges in navigating a foreign health system. One woman spoke about how abortion is illegal in her home country and the trepidation she experienced when seeking abortion care in Australia, not knowing the legal framework that permits abortion, especially at a higher gestational age, *“My culture it’s just a taboo to terminate pregnancy… to have an abortion … She [GP] said*,* ok*,* so you have this option if you want to go along with the pregnancy or if you want to terminate the pregnancy and so I didn’t have to ask about the option*,* if there was an option for termination possible here in Australia… it’s not legal at home” (P13).*

#### Geographical location as determining factor in timely care

Rural and regional women described additional challenges accessing specialist health services locally. Women with the resources to travel to access care were able to do so but were dependent on an abortion provider who was willing to perform an abortion over 20 weeks gestation, *“I was willing to travel to bloody the other side of Australia if I could” (P8).* Women living in rural and regional areas described needing to travel to a metropolitan city, arrange accommodation and transport, with some also having to arrange childcare. As one woman noted, *“There’s not much on offer” (P16).* Another participant spoke about the difficulty trying to arrange a referral for an ultrasound while maintaining confidential care in her rural community and sought an alternative GP to manage her abortion request, which delayed her abortion care even further *“I called around to about three other clinics actually to get a GP appointment…and because I’m not*,* I’m not a patient of those clinics it was also delayed (P14)”.*

### Navigating the complex abortion labyrinth

Access to abortion services and information relating to access was cited as a primary theme for delays in timely care. Participants cited service delays, lack of accessible health information and multiple referrals to abortion services as barriers to time-sensitive abortion care.

#### Service delays interrupting time-sensitive care

Primary care access was cited by participants as a factor in their delayed access of abortion care. Many experienced significant wait times to see a GP to get a referral for a pregnancy dating scan and a referral to an abortion provider. As one woman noted, “*To get a GP appointment*,* it took a week and a half just for the GP appointment to get a scan. So that I guess that’s a fairly big barrier” (p14).* Another participant described a desire to see her regular GP and wait time associated with getting an appointment, *“Yes*,* in terms of GPs*,* it was hard to get appointments. And because I wanted to speak to my regular GP*,* so it was hard” (P8).* Others faced additional barriers when sourcing a new GP, in fear of being judged by their regular GP for having an unplanned pregnancy, “*I was really quite embarrassed and not using like a local GP so I really didn’t want to do that” (P17).*

#### Access to health information

Women in this study described being proactive in familiarising themselves with services available to them, “*There’s not much information online either. I’ve got to be honest because everything just speaks about 12 weeks. There’s not much information about surgical abortion… I think because it’s just not known…it’s just an unknown topic. I would not know anything if I didn’t… If I didn’t research myself” (P1.).* Most participants in this study had never experienced an unplanned pregnancy before and had to guide themselves through the process, “*I really didn’t know anything about abortions before having to get one. That’s not something I’ve sort of familiarized myself with” (P16).*

#### Women seeking abortion shuffled between services

Women being shuffled or referred to multiple services in the context of access uncertainty, was a prominent theme due to the range of gestational age limits and capacity issues across the sector, “*I think this time was a little bit harder for me because it was more of the uncertainty of whether I was able to actually get it done or not” (p2).* Many women described having multiple referrals before being accepted for care,

*“Before*,* I initially contacted Royal Women’s*,* I spoke to a few other clinics in my area*,* and all of them*,* they actually rejected me*,* because I was more than 13 weeks pregnant. Which was really*,* it was traumatising. It was scary… I had actually met up with two GPs. And about*,* I would say*,* five*,* five clinics (P8)”.*

Participants noted how going between several services can lead to emotional distress and additional delays in accessing time-sensitive care.

#### Patient experiences with health care providers

Many of the participants in this study had both positive and negative experiences with health care providers, from the time of pregnancy diagnosis to accessing an abortion at a public hospital. Participants repeatedly shared feelings of being fortunate to be able to access a service at all, speaking to the precarious nature of abortion care over 20 weeks and the uncertainty of receiving support from a health care provider (HCP). Women described how positive experiences with healthcare providers was enabling,

“*She [GP] was really helpful. She directed me straight to the Royal Women’s which I had no idea that that was the facility in Victoria that will help in this situation. So that was really helpful. She … had that information and was really quick to give it to me. And then when I saw the Royal Women’s the doctor in the emergency department was really helpful’ (P12).*

Unfortunately, many had negative experiences with a health care provider and obstructed safe and timely care,

*“I did have a slight issue with my regular GP. A comment was kind of made in regards to abortion. And that’s why unfortunately*,* it’s been delayed out for as long as it has…my GP didn’t understand*,* she actually said that abortion was murder…Something you don’t really want to hear” (P2).*

### Enabling safe, timely, holistic, and inclusive care for all

A significant element of safe abortion care for women in this study was receiving non-judgmental, holistic, quality of care by a multidisciplinary team. Despite systemic barriers such as wait times for community scans, GP visits and appointments at a public hospital, women highlighted the importance of feeling safe and well-cared for when accessing an abortion over 20 weeks gestation.

#### Supportive providers

Overall, women spoke about the impact of non-judgmental support received, either personally or with a healthcare professional and how that reassured their decision making,

“*You know*,* I was really surprised with how supportive and not horrific I was made to feel about it. Like I really*,* I don’t know what my*,* what my expectation was*,* but I really felt like I was an absolutely awful person. But yet everyone had been really good about it. It was lovely” (P17).*

Others had expectations of being judged or mistreated but felt supported, free from judgement which facilitated a holistic safe abortion care experience,

“I just had a lot of support through them [hospital multi-disciplinary team] as well and I didn’t feel judged at all for my decision… I kind of just… it just felt like nice knowing because I’ve been so hard on myself about the whole thing. Just nice knowing that there were people there who were I felt like weren’t looking at me looking down at me for what I was doing” (P14).

#### Gratitude for access

Participants in this study recognised how limited abortion services over 20 weeks in Victoria were and described feeling fortunate for being able to access care, despite the barriers they experienced, *“I just feel like I’m one of the lucky ones” (P2).* Participants noted how feeling lucky to find a service in time to have an abortion meant there were others who were less resourced, health literate or proactive in finding a service to accommodate their care, as well as patients who couldn’t find a provider, “*I’m really lucky because I know there’s other people in my situation who wouldn’t have known how to navigate the system. And I feel like I’m quite lucky I was able to advocate for myself” (P8).* Many participants were unsure if they would be able to find a provider that would offer care over 20 weeks gestation.

## Discussion

This study aimed to give voice to the lived experiences of women who accessed abortion care at 20 weeks and over at the Royal Women’s Hospital, Melbourne, the sole provider of abortions at advanced gestations for non-medical psychosocial reasons in the state of Victoria, Australia. Women disclosed their experiences of accessing abortion care and the barriers and psychosocial contexts that delayed their care. The findings from this study reveal the complexity of the abortion-seeking process, navigating a complex health system where access to abortion care and relevant health information is not guaranteed in an Australian setting. The women in this study gave voice to the emotional, psychological, physiological, and environmental factors that delayed their abortion care, such as decision-making ambivalence, pre-existing social stressors like financial hardship, and experiencing a late diagnosis of pregnancy, making an abortion procedure earlier than 20 weeks gestation impossible.

It is well established that women seeking abortions over 20 weeks gestation often experience complex social issues such as family violence, financial hardship, limited support, and may also experience isolation during their pregnancy decision making [4, 21, 22]. Women with low educational attainment, financial hardship, unemployment, needing to travel for abortion care are disproportionately impacted by gestational age limits [7]. Findings from this study concurs with existing research that barriers to abortion access over 20 weeks gestation remains a public health challenge and continues to place additional burden on women’s lives who have pre-existing social stressors, making the abortion-seeking process even more challenging [11, 14, 22].

There is a dual hypothesis from this study. Firstly, through a structural violence lens, the focus can move beyond the individual circumstances and help us to understand how vulnerabilities intersect to perpetuate barriers to abortion access [23, 24]. Our study shows that there is and will continue to be a need for advanced gestation abortion access for women and pregnant people for as long as structural violence against women exists within our cultures and societies. Structural inequalities continue to exist for women, intersecting with physical, psychological health and the economic lives of women [25]. The embedded nature of violence against women in our society and the consequences to women’s wellbeing are not easily solved by improving access to abortion but our study highlights the ongoing need for multi-disciplinary, holistic, socio-medical models of care.

Secondly, our study hypothesises the other major barrier to abortion care over 20 weeks for non-medical reasons, is the need for increased access to earlier abortion and reproductive care services in Victoria. By identifying a gap in services in regional and remote areas, developing a workforce through training and education, improvements to local and time-sensitive care can be made. A lack of access due to restrictive gestational age limits, limited providers or lack of providers willing to perform abortions over 20 weeks impact the experience of women and pregnant people with an unplanned or unwanted pregnancy, often requiring women to travel to access abortion care over 20 weeks gestation. A third of the patients in this study were from regional or remote areas and had to travel hundreds of kilometres to access health care. Lack of local abortion services at earlier gestations and the need to travel are cited as primary reasons for delayed presentations and access to abortion care internationally [26, 27].

In keeping with existing literature, we found that women seeking abortions over 20 weeks are faced with insufficient information from health care providers, referral delays and/or not knowing where to go to access support [28]. Equitable and time-sensitive access to abortion care can be a universal challenge and remains an ever-changing landscape for women facing an unplanned or unwanted pregnancy, as evidenced by the post-Roe era in the U.S [23]. State restriction and gestational age limits, identifying a provider, financial hardship, and the need to travel to access care are cited as primary reasons for disrupted abortion access in the U.S [23]. Universal access to abortion is limited and impacts women and pregnant people who may not have the resources or capacity to travel long distances to access care, reinforcing the idea of the ‘postcode lottery’ and disempowering reproductive choice for those outside of the catchment area where health care is available [2]. In the Australian context, the 2023 Australian senate inquiry identified national barriers to universal access of abortion care and recommended that terminations should not only be legal, but are also safe, affordable, and widely accessible and free for all its residents [2].

Health information related to reproductive health and abortion care is reasonably accessible in Victoria through pro-choice GPs and online resources such as 1800MyOptions, a service of Women’s Health Victoria. However, when asked directly, many participants in this study were not aware of 1800MyOptions, the state-wide abortion and contraception database and triage service. Many surgical abortion services in Victoria decreased their capacity and reduced gestational age limits, particularly as a result of COVID-19. This has had a profound effect on abortion availability across the State, leading to confusion about where women can access services and seek support. COVID-19 exacerbated pre-existing barriers to abortion access and created a disproportionate impact on some of the most vulnerable and disempowered women in society [1]. This confusion is experienced by women seeking abortion care and by health care providers who want to support women trying to navigate the complex abortion labyrinth [9]. Victorian abortion services vary drastically in gestational age limits, capacity limitations, and formal ultrasound requirements, leading many women in this study being referred to multiple abortion services before being accepted for care at RWH. Almost half of the women in this study were born overseas, held temporary visas and spoke English as a second language. Navigating a foreign health system while attempting to access time-sensitive care was an additional barrier for many women in this study. The lack of clear, easily accessible and accurate information about abortion services and availability is a barrier to obtaining abortion care [29].

The varied backgrounds and experiences of participants in this study demonstrate that there is no one ‘type of person’ who has an abortion. Findings from this study paints a picture of individual experiences specific to a subset of psychosocial complexities that compound the contention of pregnancy decision making. There are themes and similarities throughout this study, but individual influences and circumstances are unique and highlight the complexity and potential vulnerability of individual’s lives while also navigating unplanned or unwanted pregnancy.

A US study examined demographic characteristics of 272 women seeking abortion care across 16 abortion facilities across the U.S. and found that over a third of women seeking abortion care over 20 weeks were black, less than a fifth were in their teen years and over two thirds were already mothers [22]. There is no national abortion register or database that accurately records this health metric which impedes accurate and reliable abortion rates [30]. Findings from this study are similar to data from the U.S [22]. Almost half of participants were born overseas and non-white, more than half were under thirty years old (participants under 18 years old were excluded in the criteria selection) and half were already mothers. International students and seasonal farm workers are a particularly vulnerable group, with statistics from the Australian Government showing that more than 70% of all health insurance claims for pregnancy-related treatment for all international students and their dependence occur within the first 12 months. This group are Medicare ineligible and are often excluded from private-health insurance cover due to a 12-month waiting period for non-emergency pregnancy related services, which came into effect in 2011 [31]. Prior to the ‘Overseas Health Cover Deed’ coming into effect in 2011, statistics were provided to the government outlining that up to 73% of all claims for pregnancy-related treatment for all international students and dependants occurred within the first 12 months of their arrival to Australia [31]. The majority of female international students are young, adjusting to a new life, new culture and without their usual support systems and are or are becoming sexually active. This poses a heightened probability of an unplanned pregnancy for international students, who are then seeking to navigate a foreign health system.

Almost half of the women in our study were experiencing pregnancy for the first time and were unaware that they were pregnant until a later gestation and therefore unable to access abortion care earlier than 20 weeks’ gestation. Late diagnosis of pregnancy is a universal phenomenon in international literature relating to abortion over 20 weeks gestation [11, 21]. Women who were unaware of their pregnancies experienced no signs or symptoms relating to pregnancies, leading to a late diagnosis, or thinking they were earlier in their pregnancy when confirmed [4, 32]. The term ‘unperceived pregnancy’ refers to women or pregnant people not recognising their pregnancy before 20 weeks gestation [4]. Existing research proposes that the pregnant person either experiences a psychological phenomenon of disassociation where the pregnancy is denied as a subconscious psychological protection or that they experience a physiological phenomenon of an asymptomatic pregnancy [4]. The British Pregnancy Advisory Service is the leading British abortion provider, identified the three most common reasons given for delayed detection of pregnancy were irregular periods, using contraception during conception and continuing to have a menstrual like bleeding [11].

Health literacy does not reflect a person’s general intelligence or educational literacy. Participants in this study were predominantly educated past high school yet sexual health literacy goes beyond general education. Limited access to reliable reproductive health information reflects the ‘otherness’ of abortion within the health care system. Women’s health literacy reflects the quality and access to sexual health information, bodily attunement and capacity to navigate a complex health system. Safe abortion care is more than the medico-legal safety of a procedure, it speaks to the safety of an environment that offers patients a non-judgemental, timely and inclusive model of care [7]. Positive abortion experiences begin in the community, where women interact with primary health care providers or with the state-wide triage services like 1800 MyOptions who can provide up-to-date knowledge and guidance to women seeking abortion care in a transparent and non-judgemental manner [33].

Feelings of isolation were a common experience for participants in this study, as evidenced by the high proportion of participants that concealed their pregnancy. More than half of the study participants chose not to share their pregnancies with anyone, including partners, family members or friends in fear of stigma, judgement or having their decision influenced by others, also known as reproductive coercion. Reproductive coercion is typically perpetrated by men against women in the context of fear and control [34]. Women anticipated having their bodily autonomy compromised by others and therefore chose to ‘go it alone’. Women in this study cited fear of being misunderstood or their relationships being impacted if they were to disclose their pregnancy and decision to access abortion care. For many participants, concealing a pregnancy resulted in relationship ramifications as women withdrew socially from family and friends, enduring feelings of shame and guilt. These findings support existing research, suggesting an anti-abortion rhetoric and reproductive injustice that reinforces feelings of guilt, shame, coercion, lack of consent and ultimately a lack of bodily autonomy [35]. Women and pregnant people who access abortion may internalise anti-abortion rhetoric which conflates the role of shame and guilt in a person’s abortion decision-making [35]. People seeking abortion may also perceive discrimination or judgement for accessing an abortion or have received discrimination in the process of obtaining an abortion, which contributes to negative feelings such as shame and guilt [36].

Pregnancy decision making and ambivalence are known causes for delayed access to abortion care from published research [22, 32, 37]. This is consistent with the results of our study which found that ambivalence was a common experience and many women found it difficult to make a decision regarding their unplanned or unwanted pregnancies. Almost half of participants experienced uncertainty at some point in their decision to proceed with an abortion. The choice to have an abortion was immediate for some but for others, they expressed the need for time and the opportunity to make informed decisions. There is an important distinction between complex decision making and ambivalent decision making, where ambivalence is assumed to reflect maternal attachment to the pregnancy [38]. This study highlights the complex psychosocial context that can influence pregnancy decision making rather than maternal attachment to pregnancy.

### Strengths and limitations

A strength of this study was that ACS is the sole provider of abortion care over 20 weeks gestation in the state of Victoria and therefore, captured the abortion seeking population for non-medical and psychosocial reasons at this gestation in the state. A limitation of this study was that not all women and pregnant people seeking abortion care were appropriate to recruit. Therefore, there is limited representation due to the inclusion criteria to represent the entire scope of women and pregnant people accessing abortion services over 20 weeks gestation, such as women and pregnant people under 18 years old. An additional limitation was the time-sensitive nature of the recruitment process, women were recruited once their abortion care was booked, which left limited time to set up and complete interviews prior to their surgical procedure.

This study does not focus on the provider experience of abortion over 20 weeks but is part of a wider study that highlights the experiences of abortion providers facilitating abortion care over 20 weeks at RWH [9]. It is essential to understand the barriers and enablers of abortion care both from the patient and provider perspectives to create enabling health systems and environments for abortion care.

### Implications for policy and practice

Additional research is necessary to understand both the incidence and the lived experiences of women and pregnant people who attempted to access abortion care over 20 weeks but were unable to do so. This could offer further insights into the barriers and enablers to abortion care are, from those who were unable to access care at this service. Data relating to an Australian ‘turnaway’ cohort could provide further evidence to support policy reform and ultimately the provision of comprehensive, responsive, and non-judgemental abortion services. Women seeking abortion care for psychosocial reasons is a small population, yet they face considerable stigma, as well as barriers to local and time-sensitive access to reproductive care.

De-centralising reproductive and sexual health care across Victoria could enable earlier abortion care, limiting travel and financial burden many women face when travelling to metropolitan areas to engage with abortion care. Many women in this study reflected on their own experiences and advocated for systemic change. Moreover, women interviewed advocated to support other women who had or will have similar experiences to them, wanting to contribute their voice for abortion reform. Initiatives in Victoria such as the implementation of community sexual and reproductive health hubs and the clinical champions project (led by RWH) aims to develop the wider abortion and contraception service system through education and clinical training to regional workforces to meet the needs of their local communities [39].

## Conclusion

The findings of this study highlight the psychosocial reasons women seek abortions over 20 weeks gestation and the complexities of women’s experiences accessing abortion care. These factors may include financial hardship, relationship breakdown, family violence, perceived or experienced stigma leading to concealed pregnancies, navigating the abortion health care system and needing to travel to access an abortion. Women and pregnant people experiencing an unplanned or unwanted pregnancy require time, compassionate holistic care, time-sensitive and reliable health information to allow for informed decision making. There is a need for greater abortion service access including comprehensive, responsive, and destigmatising services to ensure abortion care at advanced gestational age is available when, where and for whom it is required.

## Data Availability

No datasets were generated or analysed during the current study.

## Abbreviations

ACS: Abortion and Contraception Service
AOD: Alcohol and Other Drugs
FV: Family Violence
GP: General Practitioner
HCP: Healthcare Provider
MH: Mental Health
PIP: Partner in Pregnancy
RWH: Royal Women’s Hospital
TOP: Termination of Pregnancy
